# *Mycobacterium fortuitum* infection of the hip joint: a case report and literature review

**DOI:** 10.3389/fimmu.2026.1762789

**Published:** 2026-03-23

**Authors:** Wei Zhang, Chenfeng Zhang, Kun Shan, Shufang Liu, Jiahao Hao, Minghui Song, Cuiying Zheng, Yiran Zhao, Weili Gao, Hong Zhang, Yumei Guo, Lijie Zhang

**Affiliations:** 1 Department of Laboratory Medicine, Baoding First Central Hospital, Baoding, China; 2 Department of Laboratory Medicine, Hebei Medical University Third Hospital, Shijiazhuang, China; 3 Hebei Key Laboratory of Intractable Pathogens, Shijiazhuang Center for Disease Control and Prevention, Shijiazhuang, China; 4 Department of Laboratory Medicine, The First Hospital of Yongnian District, Handan, China

**Keywords:** 16sRNA, hip joint infection, mycobacterium fortuitum, non-tuberculous mycobacteria, rapidlygrowing non-tuberculous mycobacteria

## Abstract

Osteoarticular infections caused by *Mycobacterium fortuitum* are exceedingly rare and often result in delayed diagnosis due to nonspecific clinical manifestations. We report a case of septic arthritis of the hip in a 47-year-old man, in which *M. fortuitum* was identified from synovial fluid by 16S rRNA gene sequencing (99% identity to GenBank reference sequence CP011269.1). Upon definitive diagnosis, antimicrobial therapy was adjusted to intravenous azithromycin and levofloxacin combined with oral trimethoprim-sulfamethoxazole (TMP-SMX). Following clinical improvement, treatment was transitioned to oral azithromycin, levofloxacin, and TMP-SMX for 12 months. At the 18-month follow-up, the patient remained in complete remission without recurrence. This case highlights the critical role of 16S rRNA sequencing in diagnosing non-tuberculous mycobacterial infections and informs therapeutic strategies for similar rare infections.

## Introduction

*Mycobacterium fortuitum*, a rapidly growing non-tuberculous mycobacterium (NTM) classified within Runyon Group IV, produces visible colonies on solid media within 3-5 days. Ubiquitous in soil and dust environments, it is among the most frequently isolated environmental NTM species (1). Infections are typically associated with trauma, surgical procedures, or iatrogenic interventions, particularly among immunocompromised hosts (2). Although the global incidence of NTM infections is rising, osteoarticular involvement remains relatively rare but has gained increasing clinical attention (3). In recent years, M. fortuitum has been increasingly recognized as a pathogen in osteoarticular infections. While its role in prosthetic joint infections (PJIs), including those involving the hip, is well documented (4, 5), primary infection of a native hip joint—in the absence of prior arthroplasty, intramuscular injections, or definitive trauma—remains scarcely reported in the clinical literature. Herein, we describe a case of primary *M. fortuitum* hip infection in an immunocompetent patient with no history of joint replacement or significant trauma. Notably, the patient had undergone acupuncture prior to symptom onset. Through this case and a review of the literature, we aim to enhance clinical awareness of atypical NTM osteoarticular infections and highlight that even minor invasive procedures should be considered potential portals of entry.

## Clinical data

**Figure 1:**
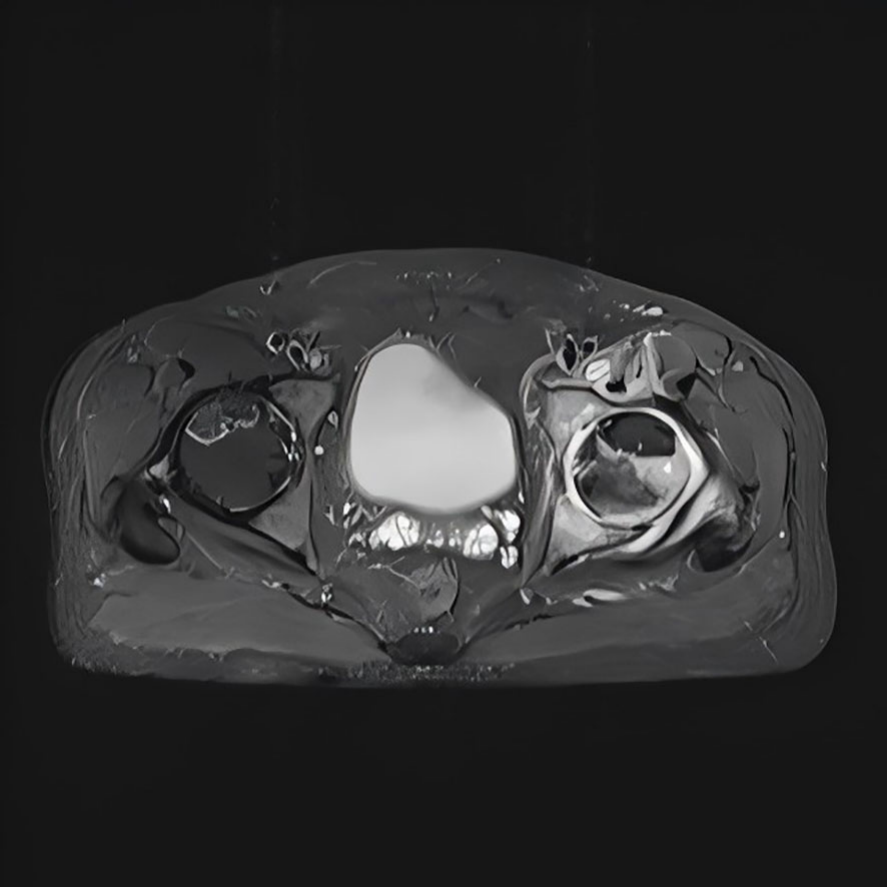
Magnetic resonance imaging (MRI) of the left hip joint demonstrates multiple areas of bony destruction involving the femoral head, neck, and acetabulum, with associated marked edema in the iliac fossa and adjacent soft tissues.

**Figure 2:**
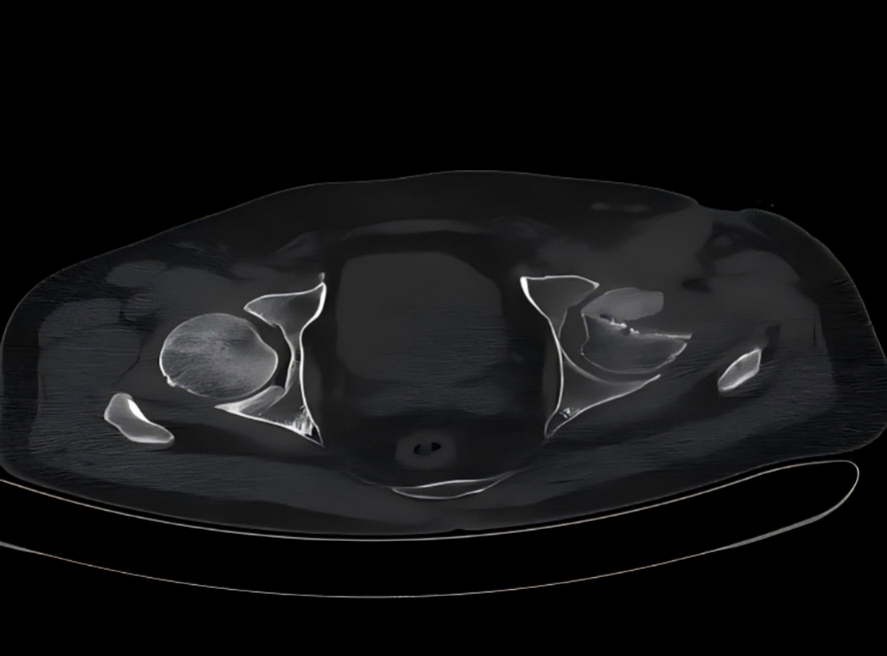
Computed tomography (CT) of the left hip joint reveals proximal bony destruction involving the femoral neck, consistent with chronic osteomyelitis.

A 47-year-old man from Shijiazhuang presented with a 3-month history of intermittent fever, 2 months of left buttock pain, and 20 days of progressive pain with restricted mobility. Pre-admission treatments for presumed lumbar disc herniation included manual spinal manipulation, acupuncture, and circulation-promoting therapies, supplemented by self-administered antipyretics with minimal relief. Except for the previously mentioned acupuncture, the patient denied any history of gluteal intramuscular injections, joint surgery, trauma, or other invasive procedures. On July 10, 2014, he presented to our hospital, and empirical therapy with cefamandole was commenced. After admission, synovial fluid from the left hip joint was aspirated for bacterial culture. During exploratory surgery of the left hip joint, a large volume of yellowish exudate was observed, accompanied by destruction of the bone at the femoral head–neck junction. The surgical field revealed exudate and granulation tissue, with partial loss of articular cartilage. Specimens of exudate and granulation tissue were collected for bacterial culture and histopathological examination. Thorough debridement was performed, followed by repeated irrigation with hydrogen peroxide, dilute povidone-iodine, and saline. One catheter was placed for continuous irrigation and another for drainage. Postoperatively, continuous negative-pressure irrigation using gentamicin sulfate solution was applied. The patient’s hip pain and restricted mobility improved relative to his preoperative condition. However, fever persisted. Magnetic resonance imaging (MRI) of the left proximal femur revealed multiple areas of bone destruction involving the femoral head, neck, and acetabulum, accompanied by significant swelling of the iliac fossa and surrounding soft tissues (**Figure 1**). Computed tomography (CT) of the left femoral neck revealed proximal osteolytic lesions with cortical erosion and periosteal reaction (**Figure 2**), consistent with chronic osteomyelitis. The patient was previously healthy, with no prior history of chronic diseases such as hypertension or diabetes.

### Laboratory findings

The patient’s laboratory tests revealed markedly elevated inflammatory markers: C-reactive protein (CRP) at 88.2 mg/L, Erythrocyte sedimentation rate (ESR) at 119 mm/h, white blood cell count (WBC) of 12.36 × 10^9/L, and neutrophil percentage of 84.01%. The red blood cell count was 4.25 × 10^12/L, and the hemoglobin level was 111.4 g/L. Plasma D-dimer, Brucella agglutination tests, rheumatoid factor panel, and tuberculosis antibody tests were negative. Acid-fast bacilli smear of centrifuged synovial fluid, performed by experienced clinical microbiologists, was also negative.

### Microbiological examination

**Figure 3:**
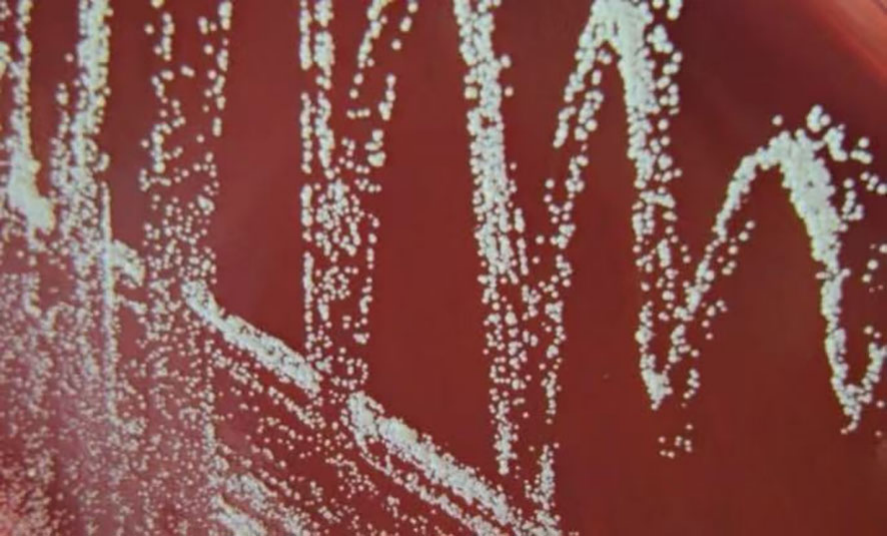
Colonies of the isolate grown on sheep blood agar for 7 days. The colonies were 2–4 mm in diameter, with a rough texture, non-hemolytic, and exhibited an orange pigmentation.

**Figure 4:**
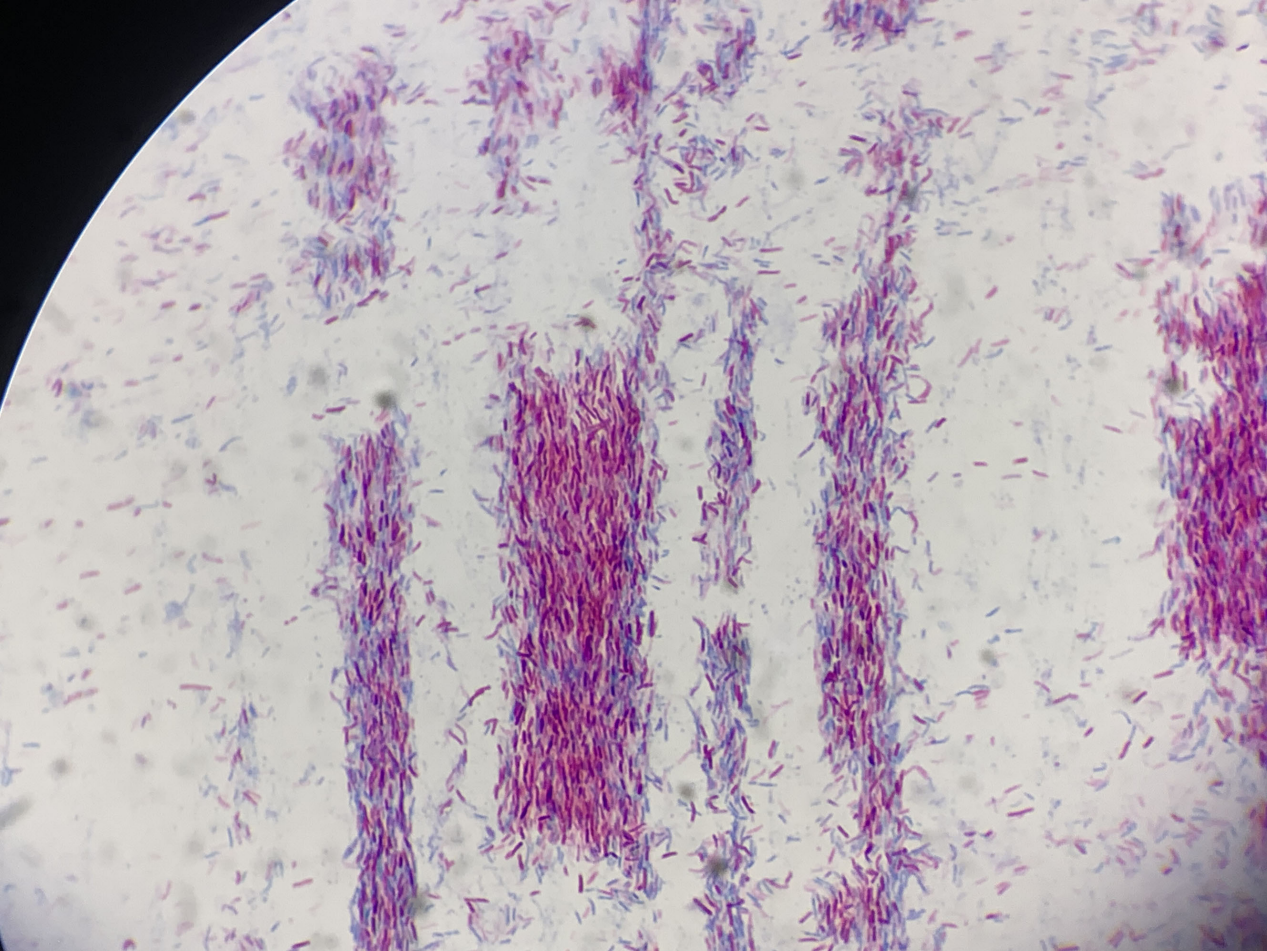
Acid-fast staining of the synovial fluid isolate. The smear demonstrates numerous slender, red-stained acid-fast bacilli, with some exhibiting a clustered arrangement (Ziehl-Neelsen stain, ×1000 oil immersion).

Blood cultures were not obtained during hospitalization. On July 10, synovial fluid was inoculated into the BacT/ALERT^®^ 3D automated culture system, which yielded a positive signal at 72 hours. Smears and subcultures were prepared on blood agar and China blue agar plates. After 2 days of incubation, numerous small, orange, rough-surfaced, non-hemolytic colonies appeared on the blood agar, which enlarged significantly by day 7 (**Figure 3**). Gram and acid-fast staining revealed the bacilli to be Gram-positive and acid-fast-positive (**Figure 4**). Based on these characteristics, the isolate was presumptively identified as a rapidly growing NTM. Accordingly, the treatment regimen was adjusted to a combination therapy consisting of intravenous azithromycin (500 mg once daily), intravenous levofloxacin (500 mg once daily), and oral trimethoprim-sulfamethoxazole (TMP-SMX; 160/800 mg twice daily).

### Bacterial identification

The isolated strain underwent 16S rRNA gene sequencing using primers 1492R (TACGGCTACCTTGTTACGACTT) and 27F (AGAGTTTGATCCTGGCTCAG), resulting in the amplification of a 1384-bp fragment. BLAST analysis of the sequence against the NCBI database revealed 99% identity with *M. fortuitum* (GenBank accession number: CP011269.1), confirming the identification. The biochemical characteristics were consistent with those of this species, including robust growth at both 28°Cand 37°C; a positive nitrate reduction test; citrate utilization; acid production from arabinose; and mannitol utilization. The strain was capable of growth in 5% NaCl and exhibited growth on thiophene-2-carboxylic acid hydrazide (TCH), para-nitrobenzoic acid (PNB), and Löwenstein-Jensen medium, all of which are characteristic of *M. fortuitum* (1).

### Treatment Outcome and follow-up

After two weeks of intravenous therapy, the patient’s body temperature normalized, and inflammatory markers, including WBC count and CRP, gradually returned to the normal range, indicating a favorable therapeutic response. Consequently, intravenous medications were discontinued, and the patient was transitioned to an oral sequential therapy regimen. Following discharge, the patient continued sterile dressing changes at a local hospital while adhering to a triple oral regimen comprising azithromycin (500 mg once daily), levofloxacin (500 mg once daily), and TMP-SMX (two tablets twice daily) for a total treatment duration of 12 months. At the 1.5-year post-treatment follow-up, no localized redness, swelling, or discharge was observed at the affected site. The patient reported no pain in the left hip and demonstrated a normal gait without limping. The range of motion (ROM) of the hip joint had largely returned to normal, and the patient has fully resumed regular work and daily activities. To date, no clinical recurrence has been observed.

## Discussion

NTM are opportunistic pathogens capable of causing infections of the skin and soft tissues, lungs, and osteoarticular system. Epidemiological studies have demonstrated a continuous global increase in the incidence of NTM infections in recent years, garnering widespread clinical attention (6). Notably, over 200 NTM species have been identified to date, approximately 60% of which are recognized as human pathogens (7). However, the majority of clinical NTM infections predominantly involve the pulmonary system, whereas musculoskeletal infections remain rare (8). This report presents a case of hip joint infection caused by *M. fortuitum*. The clinical manifestations, imaging features, and histopathological findings collectively supported this etiological diagnosis. Definitive identification was based on isolation of the pathogen from synovial fluid culture and subsequent confirmation by gene sequencing. Based on these results, timely adjustment of the antibiotic regimen resulted in significant therapeutic efficacy. Notably, although direct smear examination of the synovial fluid was negative, the pathogen was successfully isolated through culture. This discrepancy suggests that smear microscopy may yield false-negative results due to limitations such as sampling scope and microscopic field coverage. These findings underscore the importance of culture methods and molecular techniques in the accurate diagnosis of NTM infections, especially when smear results are negative.

Osteoarticular infections caused by NTM are rare, often presenting with non-specific clinical features that pose substantial challenges for timely and accurate diagnosis as well as effective management. Although existing guidelines and consensus statements provide treatment recommendations (9, 10), clinical management must be individualized, guided by published cases and the specific characteristics of affected joints. To characterize the epidemiological patterns and treatment strategies for osteomyelitis caused by *M. fortuitum*, we performed a search of the PubMed database (1973–2025) using the keywords “osteomyelitis” and “*M. fortuitum*,” and identified 15 confirmed cases (see **Table 1**). The majority of patients were male (9 cases, 60.0%), with a broad age distribution; notably, individuals over 50 years old constituted the largest proportion (46.7%). The lower extremities were the most frequently affected sites (12 cases, 80.0%), likely due to their increased susceptibility to trauma or surgical procedures. Etiological analysis revealed that trauma—such as open fractures, penetrating injuries, and postoperative infections—was the predominant precipitating factor (12 cases, 80.0%), while fewer cases were linked to intravenous drug use or immunosuppression. In terms of treatment, most patients received combination antibiotic therapy, frequently involving doxycycline (6 cases, 40.0%), amikacin (6 cases, 40.0%), and cefoxitin (4 cases, 26.7%). Several cases also underwent surgical debridement and adjunctive interventions. Overall, treatment outcomes were favorable, with a reported cure rate of 80.0% (12 cases). This case is distinguished from the majority of previously reported instances by the absence of overt trauma, joint replacement, or gluteal intramuscular injections. The clinical progression was characterized by an insidious and prolonged course. Importantly, the patient had undergone acupuncture prior to the onset of symptoms. While a direct causal relationship cannot be definitively established, such percutaneous procedures may theoretically serve as a potential portal of entry for NTM. Given prior reports of *M. fortuitum* infections following intramuscular injections (26), this case underscores the necessity of considering even minor invasive procedures as potential risk factors for atypical joint infections.

**Table 1:** Summary of published *Mycobacterium fortuitum* osteomyelitis cases (1973–2025).

Reference	Country/age/gender	Pathogen	Infection Site	Underlying cause/trauma	Treatment regimen	Outcome
(11)	China/66/M	*M. fortuitum*	Right tibia and fibula (midshaft)	Open wound	Cefoxitin, Amikacin, Doxycycline (alternative agents)	RW
(12)	Spain/61/F	*M. fortuitum*	Lunate bone	Penetrating injury	Linezolid, Doxycycline	RW
(13)	Singapore/55/M	*M. fortuitum*	Right foot	Open fracture-dislocation, flap reconstruction	Cefoxitin, Clarithromycin, Doxycycline	RW
(14)	USA/10/F	*M. fortuitum*	Lunate, scaphoid, metatarsals	Plantar penetrating injury	Azithromycin, Ciprofloxacin, TMP-SMX	RW
(15)	USA/47/F	*M. fortuitum*	L5–S1 intervertebral disc	IV drug use	Amikacin, Cefoxitin, TMP-SMX, Doxycycline	NA
(16)	China/72/F	*M. fortuitum, M. abscessus*	Right knee	Post total knee arthroplasty	Doxycycline, Ciprofloxacin, Clarithromycin, Amikacin	RW
(17)	China/35/M	*M. fortuitum, M. chelonae*	Left forearm	Open fractures of radius and ulna	Levofloxacin, Amikacin, Clarithromycin	RW
(18)	USA/68/M	*M. fortuitum*	Both knees	Bilateral total knee arthroplasty	Cefoxitin, Amikacin, Ciprofloxacin, Clarithromycin, Meropenem, Linezolid	RW
(19)	USA/33/M	*M. tuberculosis, M. fortuitum*	Right ankle	Ankle sprain	INH, RIF, EMB, Doxycycline, Ciprofloxacin	NA
(20)	Israel/11/M	*M. fortuitum*	Lunate bone	Nail puncture to foot	Amikacin	RW
(21)	USA/35/M	*M. fortuitum*	Humerus	Open humeral fracture	Ciprofloxacin	RW
(22)	Japan/17/M	*M. fortuitum*	Right lower limb	Post-op after K-wire internal fixation	Hardware removal, anti-TB drugs, Ofloxacin, IL-2	RW
(23)	USA/66/M	*M. fortuitum*	Right ankle	Open dislocation fracture	Erythromycin, Minocycline	RW
(24)	USA/14/F	*M. fortuitum*	Right calcaneus	Plantar puncture injury	Ethambutol, Rifampin	RW
(25)	USA/68/F	*M. fortuitum*	Toe phalanges	Distal phalangeal fracture	Ethambutol	RW

RW, recovered well; NA, not available; INH, isoniazid; RIF, rifampin; EMB, ethambutol; TMP-SMX, trimethoprim-sulfamethoxazole; IL-2, interleukin-2.

Treatment of NTM infections remains highly challenging due to the intrinsic resistance of these pathogens to multiple antimicrobial agents, particularly conventional anti-tuberculosis drugs (27). Consequently, management of osteoarticular NTM infections typically requires long-term, individualized combination therapy guided by *in vitro* drug susceptibility testing. The 2020 guidelines from the American Thoracic Society (ATS), Infectious Diseases Society of America (IDSA), European Respiratory Society (ERS), and European Society of Clinical Microbiology and Infectious Diseases (ESCMID) primarily address pulmonary NTM infections; however, their therapeutic principles can inform the management of osteoarticular cases. Initial therapy should comprise at least two agents to which the isolate demonstrates *in vitro* susceptibility. These usually include a macrolide (e.g., clarithromycin or azithromycin) in combination with an aminoglycoside (e.g., amikacin) or a fluoroquinolone (e.g., moxifloxacin), with modifications guided by patient-specific factors (9). Macrolides are pivotal, not only inhibiting bacterial protein synthesis but also for modulating host immune responses, thereby enhancing therapeutic efficacy (28). In the present case, upon confirmation of *M.fortuitum* by 16S rRNA gene sequencing, a triple-drug regimen was administered, consisting of intravenous azithromycin, intravenous levofloxacin, and oral TMP-SMX. This treatment resulted in the normalization of body temperature, resolution of hip symptoms, and reduction of inflammatory markers (CRP and ESR) to normal levels. The patient remained free of relapse during an 18-month follow-up, achieving a favorable clinical outcome. Notably, *in vitro* susceptibility testing was not performed in 2014 due to laboratory constraints, reflecting the constraints of NTM diagnostic capabilities at the time. Nevertheless, this successful outcome underscores that empirical therapy based on international guidelines remains a viable approach for managing infections caused by rapidly growing mycobacteria, even in resource-limited settings. Furthermore, early and accurate pathogen identification is critical for effective management of NTM infections. In this case, 16S rRNA gene sequencing enabled the rapid identification of *M. fortuitum*, significantly improving diagnostic accuracy and timeliness compared to conventional culture and phenotypic methods. The early suspicion of NTM infection by clinical microbiologists, based on colony morphology and staining results (Gram-positive and acid-fast bacilli), was instrumental in prompting molecular testing and guiding targeted therapy. The integration of these diagnostic modalities substantially reduced the time to pathogen identification, securing a critical therapeutic window and ultimately contributed to the successful outcome.

This case demonstrates that *M. fortuitum*, a rapidly growing NTM, can cause deep-seated osteoarticular infections even in the absence of conventional risk factors. This finding emphasizes the necessity of including NTM infections in the differential diagnosis of chronic and refractory joint disorders. Accurate and timely pathogen identification, coupled with the prompt initiation of an effective, individualized treatment regimen, is essential for achieving favorable patient outcomes.

## Data Availability

The original contributions presented in the study are included in the article/supplementary material. Further inquiries can be directed to the corresponding authors.
